# Supplementary material for: Planning the electric vehicle transition by integrating spatial information and social networks
Source: Nat Commun. 2025 Dec 11;16:11220. doi: 10.1038/s41467-025-66072-5 (PMC12714869; doi:10.1038/s41467-025-66072-5)
Supplement: Supplementary file 1 — Supplementary Information [file 41467_2025_66072_MOESM1_ESM.pdf]

Supplemental Information

Note S1: Fitting Error on County Level

Figure 1 shows 2022 county-level adoption rates for Washington and California (excluding the 15 most populated counties) from data and simulations of State BM, County BM, and SocNet BM.

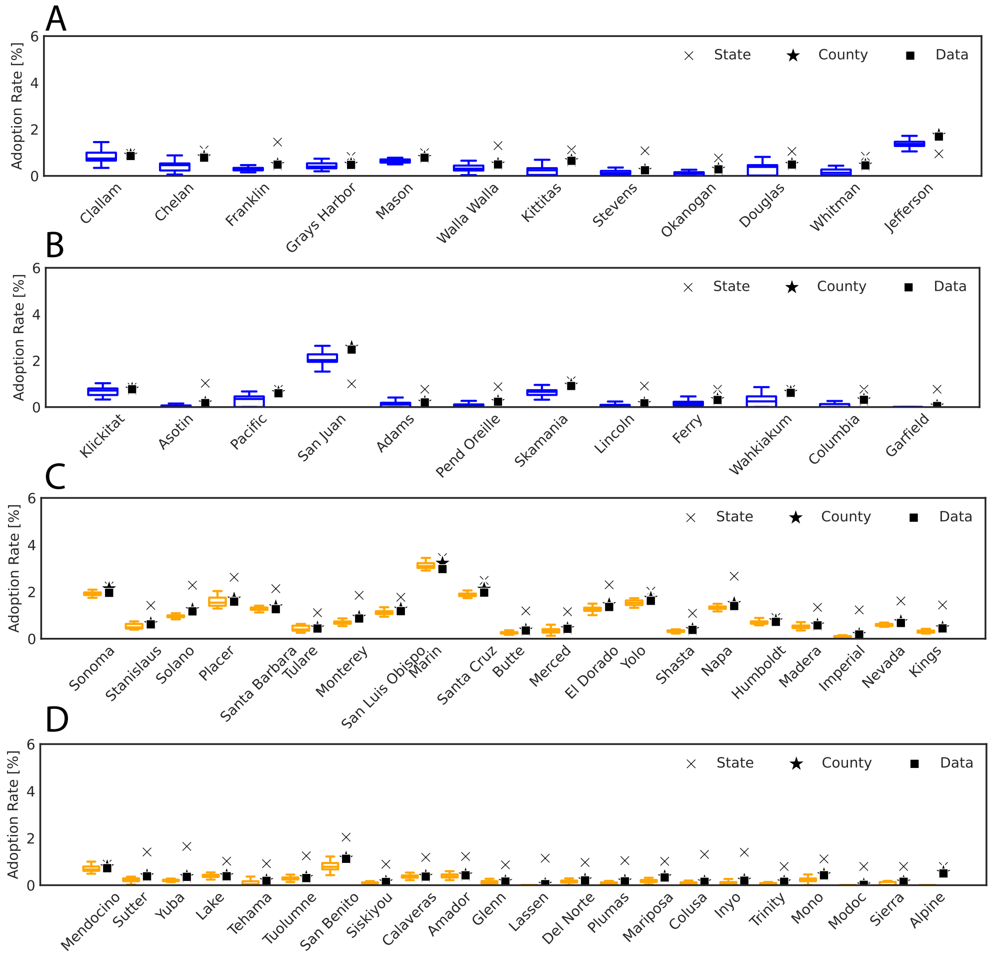

Figure 1: County-level adoption rates of data, State/County BM and SocNet BM in 2022. State/County BM is based one estimated parameter set, while SocNet BM results are obtained by averaging 10 simulations for each of three independently estimated parameter sets. The box represents the interquartile range (25th–75th percentiles) of county-level adoption rates of SocNet BM, the center line marks the median, and the whiskers indicate the minimum and maximum values. Cross markers show the adoption rate of State BM, star markers show the adoption rate of County BM, and square markers show the empirical data. (A-B) demonstrate results in Washington (blue) and (C-D) demonstrate results in California (orange).

Note S2: Fitting Error on Tract Level

Tract-level fitted adoption rates from State BM, County BM, SocNet BM, and data in 2022 are shown in Fig. 2.

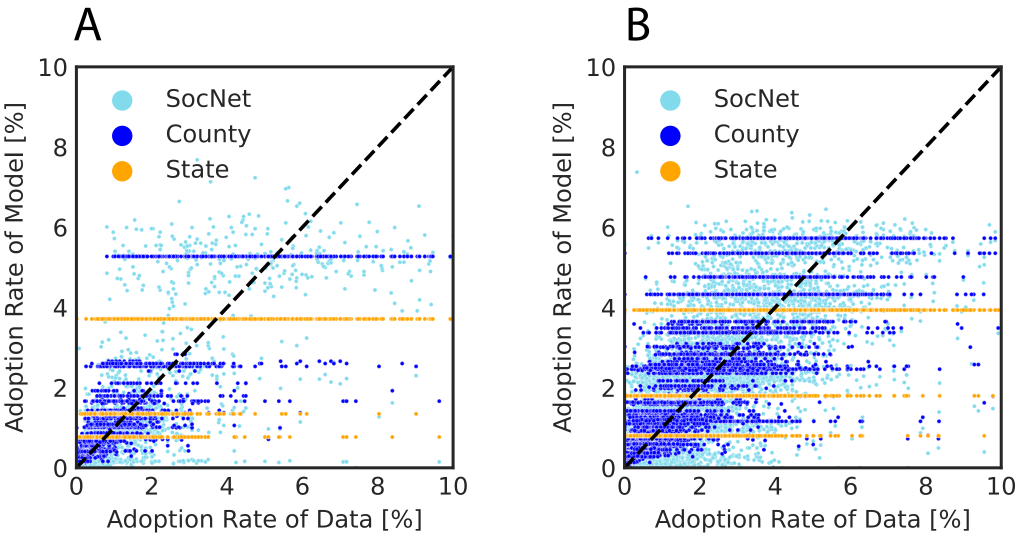

Figure 2: Tract-level adoption rate comparison between empirical data and simulations of State BM (orange), County BM (dark blue), and SocNet BM (light blue) in 2022. State/County BM is based one estimated parameter set, while SocNet BM results are obtained by averaging 10 simulations for each of three independently estimated parameter sets. Each point presents the fitted tract-level adoption rate and the corresponding empirical adoption rate. The diagonal line indicates fitted adoption rate is the same as the empirical data. (A) demonstrates results in Washington and (B) demonstrates results in California.

Note S3: Coefficients of State/County BM and SocNet BM

Table 1 demonstrates fitted p and q values from State/County BM and SocNet BM.

	p^{Low}	p^{Middle}	p^{High}	q^{Low}	q^{Middle}	q^{High}
Washington State	1.4×10^{-4}	2.6×10^{-4}	7.6×10^{-4}	0.21	0.21	0.20
Washington County	7.4×10^{-5}	1.3×10^{-4}	1.8×10^{-4}	0.23	0.22	0.22
Washington SocNet	1.5×10^{-4}	3.4×10^{-4}	4.0×10^{-4}	0.02	0.03	0.03
California State	2.3×10^{-4}	6.2×10^{-4}	2.0×10^{-3}	0.15	0.13	0.08
California County	1.4×10^{-4}	2.9×10^{-4}	8.8×10^{-4}	0.14	0.14	0.11
California SocNet	1.2×10^{-4}	4.7×10^{-4}	1.2×10^{-3}	0.02	0.01	0.05

Table 1: Median p and q values of State/County BM and SocNet BM (units for BM parameters are $year^{-1}$).

Note S4: Forecast Considering "Switching Back to Internal Combustion Engine Vehicles (ICEVs)"

Various elements—including fluctuations in fuel/electricity prices, the availability of charging stations, and changes in consumer tastes—may cause a reversal towards older technology. According to [1], approximately 46% of current PEV users in the United States may consider reverting to ICE vehicles. Our model allows for the simulation of "Switching Back to ICEVs" by assigning a likelihood that users will abandon PEVs after several years of use. We examine different scenarios by adjusting two parameters: *Year* indicates the number of years before users start to consider transitioning back to ICEVs, while *Prob* expresses the probability of this switch. For example, "$Year = 5$ and $Prob = 0.5$" indicates that after 5 years of adoption, the adopter will have a probability of 0.5 of switching back to ICEVs every year thereafter. Our settings include $Year = 5$, 10, 15 and $Prob = 0.1$, 0.3, 0.5. Setting "No Return" represents the baseline where customers do not switch back to ICEVs. Fig. 3 (A-B) demonstrates how a smaller *Year* and a larger *Prob* slows down the PEV diffusion in Los Angeles.

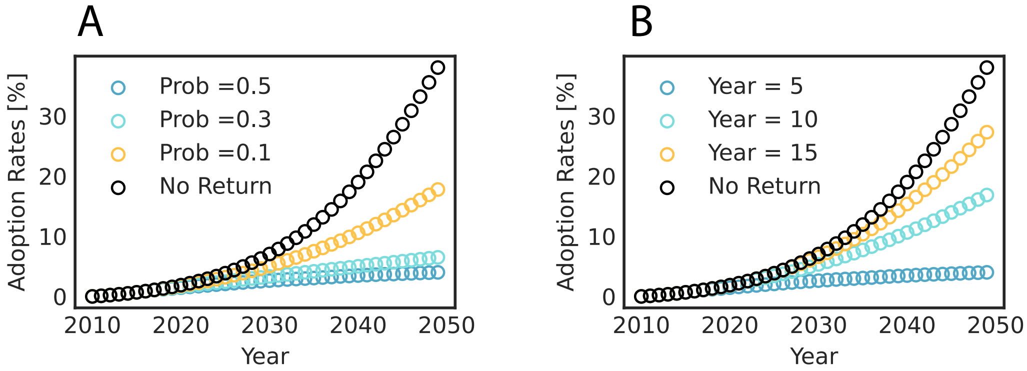

Figure 3: PEV diffusion forecast accounting for "Switching Back to ICEVs". Results are obtained by averaging 10 simulations. (A) demonstrates adoption forecast based on varying timeframes (in years) before users begin considering a transition back to ICEVs (*Year* set as 5). (B) demonstrates adoption forecast based on different probabilities of switching back to ICEVs (*Prob* set as 0.5).

Note S5: Forecasts on County Level

Figure 4 shows 2050 county-level adoption rates for Washington and California (excluding the 15 most populated counties) from data and simulations of State BM, County BM, and SocNet BM.

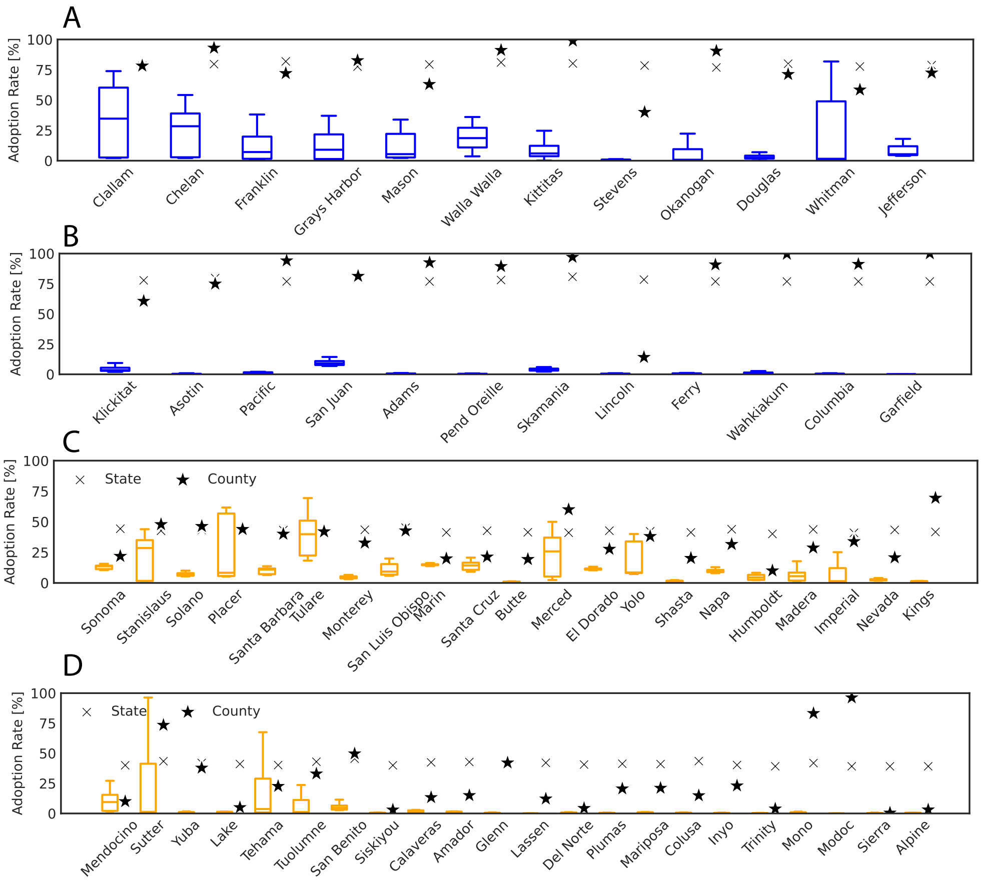

Figure 4: County-level adoption rates of data, State/County BM and SocNet BM in 2050. State/County BM is based on a uniquely estimated parameter set, while SocNet BM results are obtained by averaging 10 simulations for each of three independently estimated parameter sets. The box shows the quartiles of the county-level adoption rates of SocNet BM while the whiskers extend to show the rest of the distribution. The box represents the interquartile range (25th–75th percentiles) of county-level adoption rates of SocNet BM, the center line marks the median, and the whiskers indicate the minimum and maximum values. Cross markers show the adoption rate of State BM, star markers show the adoption rate of County BM, and square markers show the empirical data. (A-B) demonstrate results in Washington (blue) and (C-D) demonstrate results in California (orange).

Note S6: Urban Layout and Diffusion Process

The urban layout of the area directly affects the spatial and social structures of areas, which can further affect PEV adoption. This section provides an analysis of the correlation between urban spatial/social structures and adoption diffusion patterns. Regarding spatial structure, we consider the geographical autocorrelation of population density, the geographical autocorrelation of income, and the overall population density at the county level. Concerning the social structure of areas, we take into account various γ values that determine the social network structure.

In terms of spatial structure, Fig. 5 demonstrates that counties with greater population and higher geographical autocorrelation in terms of income and population density typically exhibit higher adoption rates in present and forthcoming adoption trends.

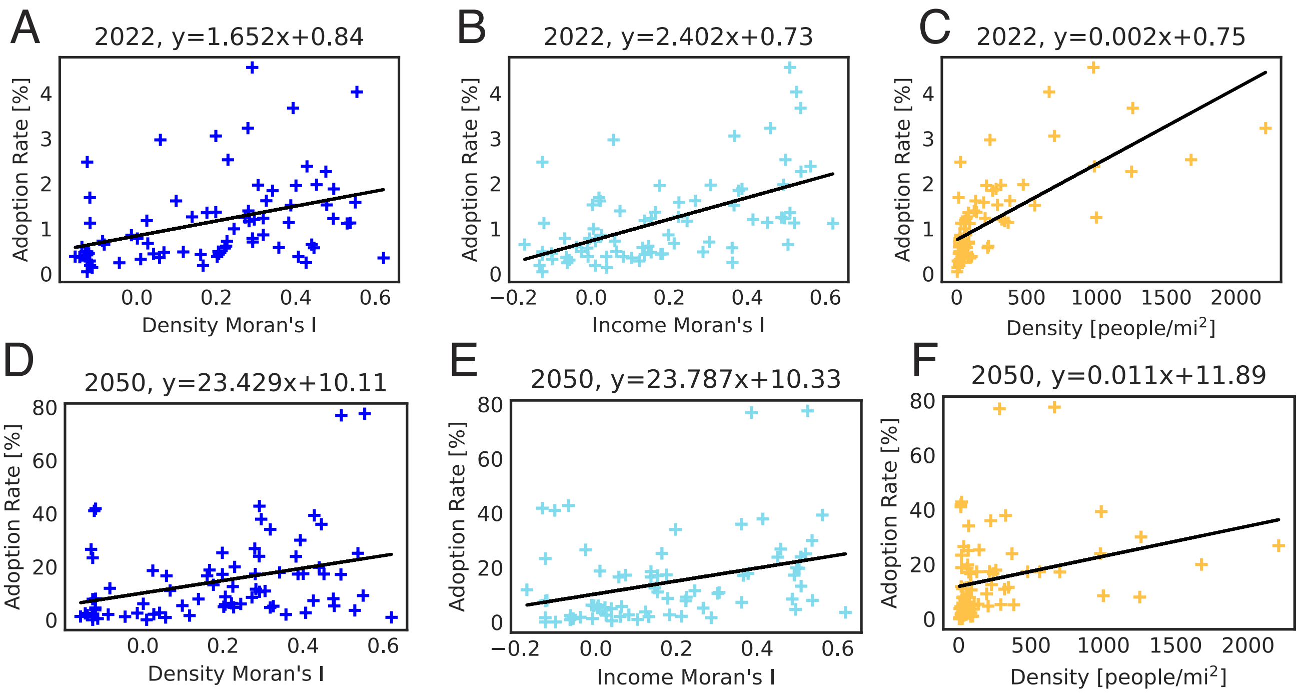

Figure 5: Spatial structure and diffusion process. Each circle represents one county in Washington and California. The solid line denotes the linear regression results of circles. (A-C) plot the county-level adoption rate and corresponding population density Moran's I, Income Moran's I, and overall population density in 2022. (D-F) plot county-level adoption rate and corresponding population density Moran's I, Income Moran's I, and overall population density in 2050.

In terms of social structure, Fig. 6 (A-C) display the Moran's I statistics for State/County BM and SocNet BM across three counties in Washington, while Fig. 6 (D-F) show the same in California. In California and Washington's most populous counties, specifically King and Los Angeles, we can identify a social network structure parameter (γ) that closely replicates the observed Moran's I statistics. Nonetheless, counties such as Santa Clara and Pierce have no γ value that can accurately replicate the empirical Moran's I statistics.

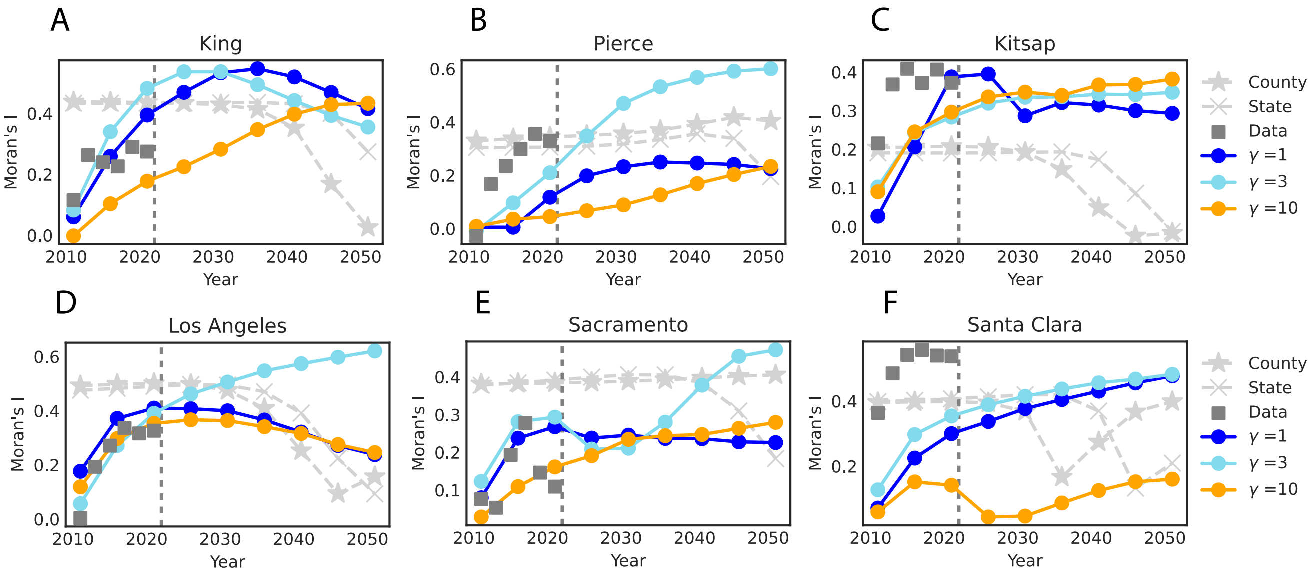

Figure 6: Moran's I statistics for State/County BM and SocNet BM in 6 counties in Washington and California. State/County BM is based on a uniquely estimated parameter set, while SocNet BM results are obtained by averaging 10 simulations. For SocNet BM, we use the network with three γs, i.e., 1,3,10. The fitting process is repeated for each γ and the median of predictions is reported. Solid lines with circle markers show the median (50th percentile) Moran's I statistics from SocNet BM. Dashed lines with cross markers and star markers show the Moran's I statistics from State/County BM; Squares show the Moran's I statistics calculated from empirical data on tract level.

Note S7: Justification of Market Segmentation

In this study, we divide the potential market based on income. Other related segmentation factors include educational background, home ownership, and housing type. In Fig. 7, we show that these three variables exhibit correlations. The figure illustrates the association of these three variables with income. Besides, with only three income groups, the fitted p and q already demonstrate significant variability due to limited data at the early adoption stage. Hence, despite the potential for more advanced applications to incorporate additional market segmentation with more socio-demographic parameters [2] or varying levels of the variables[3], we select income as the sole segmentation criterion.

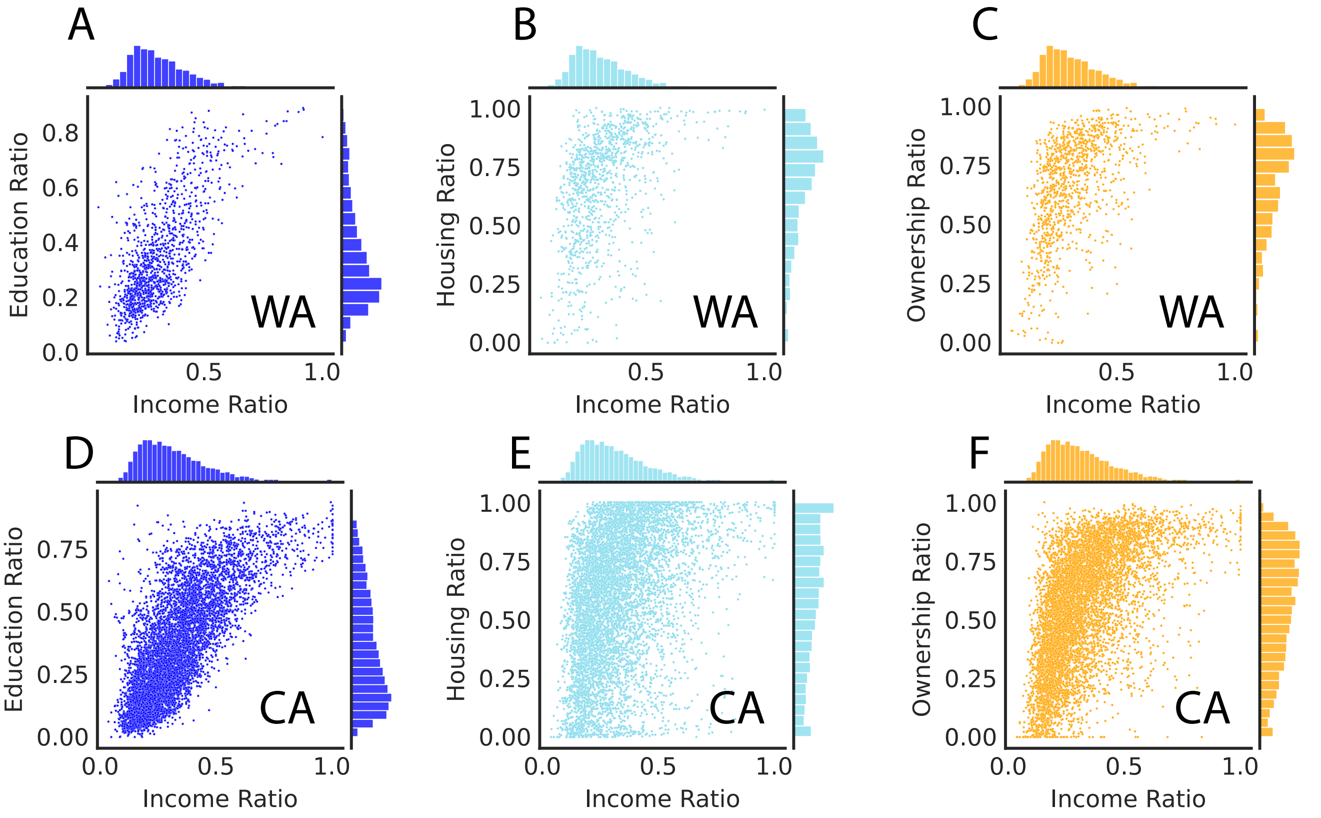

Figure 7: Correlation plots of socio-demographic variables related to adoption. The income ratio represents the normalized income relative to the highest income across all tracts. (A,D) demonstrate correlation between the education ratio and income in Washington and California. The education ratio is defined as the proportion of the population aged 25 or older who have at least a Bachelor's degree, relative to the total population aged 25 and older. (B,E) demonstrate correlation between ownership ratio and income in Washington and California. The ownership ratio refers to the percentage of owner-occupied units among all housing units. (C,F) demonstrate correlation between the housing ratio and income in Washington and California. The housing ratio indicates the percentage of detached single-family units among total housing units.

Note S8: Sensitivity Analysis on Network Size

This sensitivity analysis illustrates the impact of varying network sizes on the social network structure, adoption rate, and Moran's I for the adoption rate. We configure various network sizes by selecting expansion factors of 5, 10, 15, 25, and 100 in Los Angeles. As presented in Table 2-4, an increase in the expansion factor results in reduced network size, characterized by fewer nodes and edges. Our analysis also demonstrates how average degree, average geographical distance between two nodes, clustering coefficient, and assortativity vary across different expansion factors.

Expansion Factor	# Nodes	# Edges	Average Degree	Average Distance	Clustering Coefficient	Assortativity
5	1,189,150	3,567,441	6	21.67	0.0001	0.07
10	594,480	1,783,431	6	21.68	0.0002	0.07
15	396,364	1,189,083	6	21.66	0.0002	0.07
25	237,827	713,472	6	21.70	0.0004	0.07
100	59,453	178,350	6	21.76	0.0012	0.07

Table 2: Expansion factor and corresponding social network structure summary ($\gamma = 1$)

Expansion Factor	# Nodes	# Edges	Average Degree	Average Distance	Clustering Coefficient	Assortativity
5	1,189,150	3,567,441	6	1.95	0.01	0.65
10	594,480	1,783,431	6	1.96	0.02	0.65
15	396,364	1,189,083	6	1.97	0.02	0.65
25	237,827	713,472	6	1.99	0.03	0.64
100	59,453	178,350	6	2.14	0.08	0.62

Table 3: Expansion factor and corresponding social network structure summary ($\gamma = 3$)

Expansion Factor	# Nodes	# Edges	Average Degree	Average Distance	Clustering Coefficient	Assortativity
5	1,189,150	3,567,441	6	0.71	0.04	0.96
10	594,480	1,783,431	6	0.71	0.07	0.96
15	396,364	1,189,083	6	0.72	0.10	0.95
25	237,827	713,472	6	0.72	0.14	0.95
100	59,453	178,350	6	0.78	0.33	0.92

Table 4: Expansion factor and corresponding social network structure summary ($\gamma = 10$)

We further illustrate the impact of varying expansion factors on adoption rates and Moran's I. Figure 8 (A-C) shows that smaller expansion factors result in higher Moran's I, whereas Fig. 8

(D-F) indicates that the expansion factor has minimal effect on adoption rates. Consequently, we decide to use the smallest feasible expansion factor, which is 5.

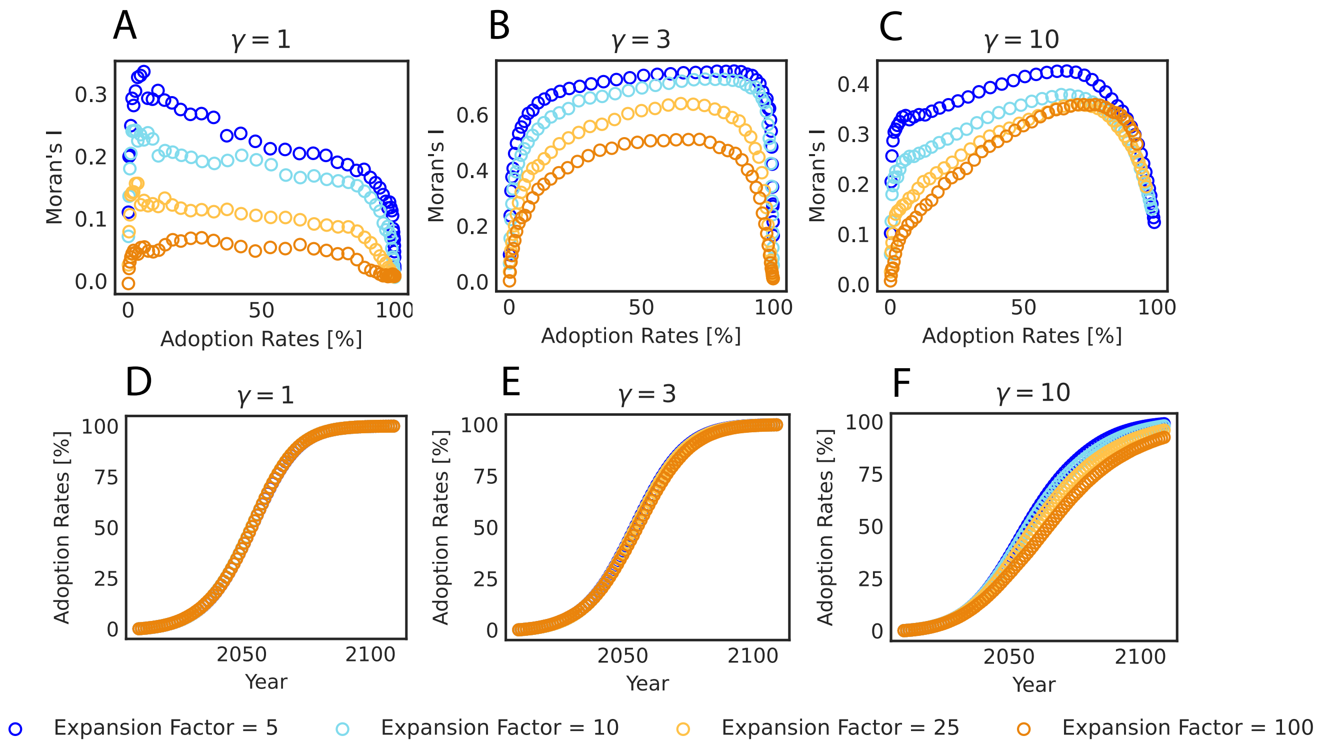

Figure 8: Network expansion factors' influence on diffusion patterns. The dots present the median results of 10 repetitions of simulation using a predefined p and q set. (A-C) demonstrate the correlation of adoption rates and corresponding Moran's I across various expansion factors (Expansion Factor = 5, 10, 25, 100) and different network structures ($\gamma = 1, 3, 10$). (D-F) demonstrate adoption rates evolution across various expansion factors (Expansion Factor = 5, 10, 25, 100) and different network structures ($\gamma = 1, 3, 10$).

Note S9: Justification of Values of γs

We calculate clustering coefficient [4], assortativity [5], the distribution of degree, and distribution of the geographical distance between nodes in Los Angeles. Table 5 and Fig. 9 show the overview of social network characteristics with different γ. We find that higher γ leads to higher clustering coefficients, assortativity, $P(k)$ exponents, and $P(r)$ exponents.

Previous research finds for the social network inferred from blogs [6], location-based social networks [7][8] or mobile phone data [9][10][11], the distribution of geographical distance between nodes follows a power law, with exponents between -1 and -2. Therefore, we explore social networks with $\gamma = 1, 3, 10$, which leads exponents between 0 to -3. Also, we set $\gamma = 10$ for the default social network for analysis as it leads to a clustering coefficient closer to empirical values in social networks.

γ	Clustering Coefficient	Assortativity	P(k) Power Law Fitted Exponent	P(r) Power Law Fitted Exponent
1	0.0001	0.07	-3	-0.3
3	0.01	0.65	-3	-2.1
10	0.04	0.96	-3	-3.0

Table 5: Social Network Characteristics with Different γ

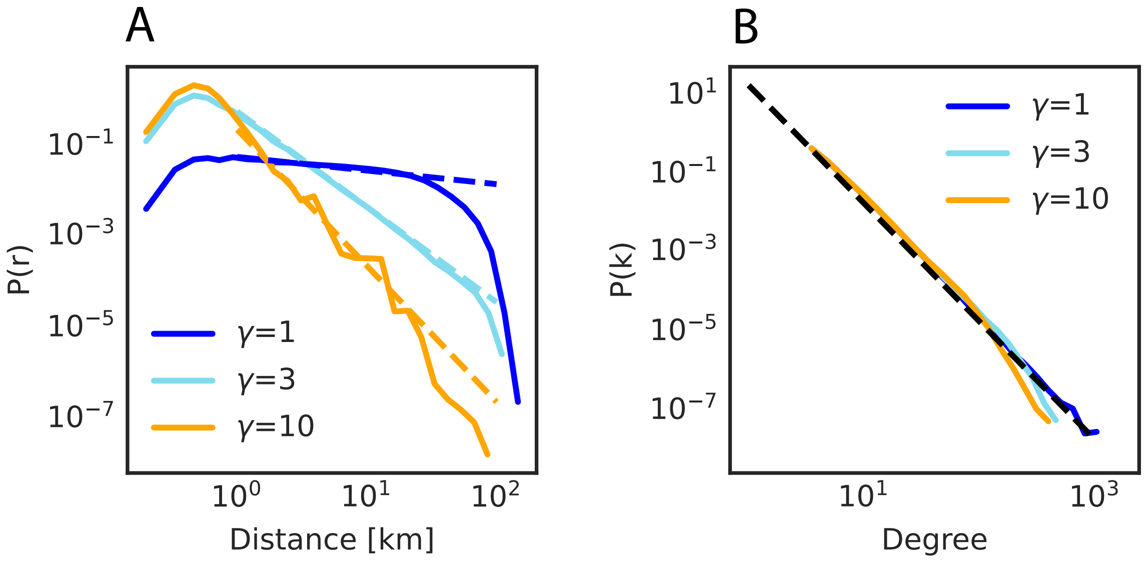

Figure 9: Distance distribution $P(r)$ and Degree distribution $P(k)$ of social network with $\gamma = 1, 3, 10$. (A) Distance distribution of various social network structures. The solid line indicates the data distribution and the dashed line with the same color indicates the fitted power law distribution. (B) Degree distribution of various social network structures. The solid line indicates the data distribution and the grey dashed line indicates the fitted power law distribution.

Note S10: Social Network Construction Process

Fig. 10 depicts the procedure for constructing a social network. Subfigures (A-C) illustrate the assignment of geographical information to nodes based on census data [12], while (D-F) illustrate the process of forming edges among nodes.

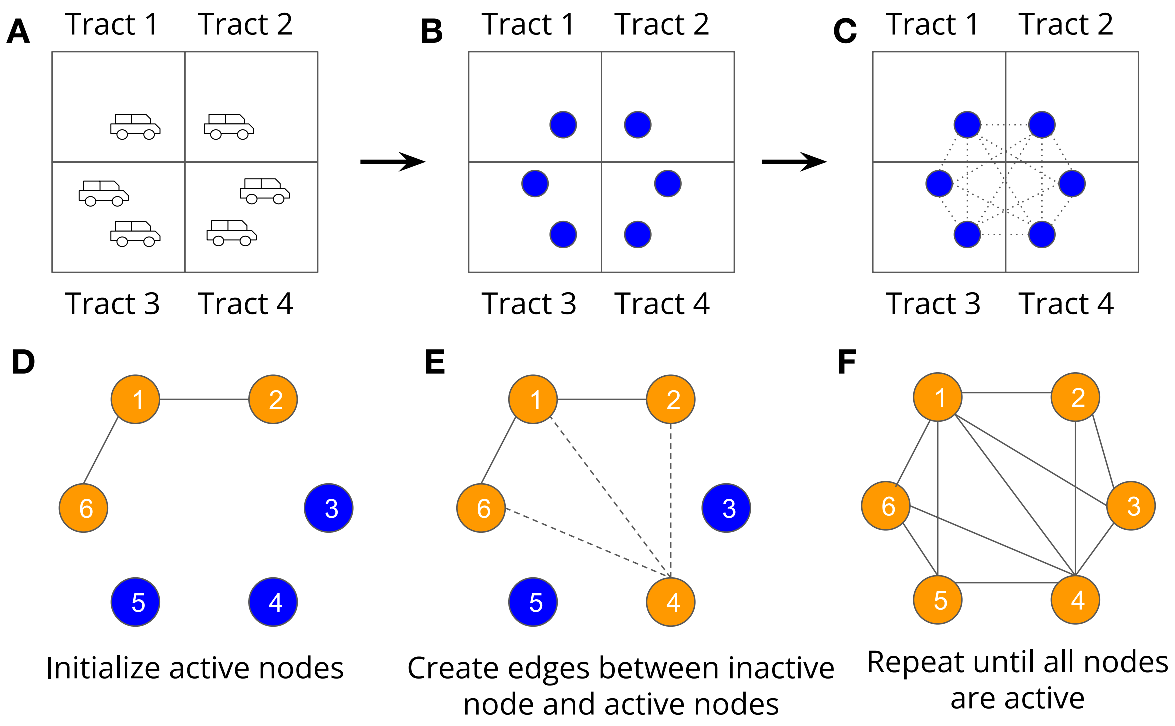

Figure 10: Social network construction process. (A) Number of vehicles in each census tract (obtained from the census tract data). (B) Each node represents one node (when the expansion factor is 1). The latitude and longitude for each node are set as centroid coordinates of its respective census tract. (C) Based on the latitude and longitude for each node, the distance d_{ij} between any two nodes, i and j, can be computed. For nodes residing within the same census tract, the distance is set as half the square root of the census tract's area. (D) Nodes 1, 2, and 6 are selected as the active nodes set, leaving nodes 3, 4, and 5 as inactive. (E) Inactive node 4 is randomly chosen, and $prob_{4\rightarrow j}$ is calculated and normalized where $j \in \{1, 2, 6\}$. For example, for node 4 and node 1, $k_4 = 0$ and d_{41} is the distance between the centroid of census tract 4 and census tract 1. Based on $prob_{4\rightarrow j}$, $m = 3$ edges are created linking node 4 to nodes in the active nodes set. Node 4 is then marked as active. This step is repeated for the remaining inactive nodes until all are active. (F) This process is executed for nodes 3 and 5 to finalize the social network.

References

[1] McKinsey & Company, "Mckinsey mobility consumer pulse." https://executivedigest.sapo.pt/wp-content/uploads/2024/06/Mobility-Consumer-Pulse-2024_Overview.pdf, 2024.

[2] J. H. Lee, S. J. Hardman, and G. Tal, "Who is buying electric vehicles in california? characterising early adopter heterogeneity and forecasting market diffusion," *Energy Research & Social Science*, vol. 55, pp. 218–226, 2019.

[3] Y. Yuan, Y. Zhou, Z. Lin, and K. Jin, "Prediction of pev adoption with agent-based parameterized bass network diffusion model," *arXiv preprint arXiv:2303.15313*, 2023.

[4] J. Saramäki, M. Kivelä, J.-P. Onnela, K. Kaski, and J. Kertesz, "Generalizations of the clustering coefficient to weighted complex networks," *Physical Review E*, vol. 75, no. 2, p. 027105, 2007.

[5] M. E. Newman, "Mixing patterns in networks," *Physical review E*, vol. 67, no. 2, p. 026126, 2003.

[6] D. Liben-Nowell, J. Novak, R. Kumar, P. Raghavan, and A. Tomkins, "Geographic routing in social networks," *Proceedings of the National Academy of Sciences*, vol. 102, no. 33, pp. 11623–11628, 2005.

[7] S. Scellato, A. Noulas, and C. Mascolo, "Exploiting place features in link prediction on location-based social networks," in *Proceedings of the 17th ACM SIGKDD international conference on Knowledge discovery and data mining*, pp. 1046–1054, 2011.

[8] E. Cho, S. A. Myers, and J. Leskovec, "Friendship and mobility: user movement in location-based social networks," in *Proceedings of the 17th ACM SIGKDD international conference on Knowledge discovery and data mining*, pp. 1082–1090, 2011.

[9] R. Lambiotte, V. D. Blondel, C. De Kerchove, E. Huens, C. Prieur, Z. Smoreda, and P. Van Dooren, "Geographical dispersal of mobile communication networks," *Physica A: Statistical Mechanics and its Applications*, vol. 387, no. 21, pp. 5317–5325, 2008.

[10] J.-P. Onnela, J. Saramäki, J. Hyvönen, G. Szabó, D. Lazer, K. Kaski, J. Kertész, and A.-L. Barabási, "Structure and tie strengths in mobile communication networks," *Proceedings of the national academy of sciences*, vol. 104, no. 18, pp. 7332–7336, 2007.

[11] C. Herrera-Yagüe, C. M. Schneider, T. Couronne, Z. Smoreda, R. M. Benito, P. J. Zufiria, and M. C. González, "The anatomy of urban social networks and its implications in the searchability problem," *Scientific reports*, vol. 5, no. 1, p. 10265, 2015.

[12] US Census Bureau, "Census bureau data." https://data.census.gov, 2023. Retrieved on March, 2023.
