# Supplementary material for: Planning the electric vehicle transition by integrating spatial information and social networks
Source: Nat Commun. 2025 Dec 11;16:11220. doi: 10.1038/s41467-025-66072-5 (PMC12714869; doi:10.1038/s41467-025-66072-5)
Supplement: Supplementary file 2 — Reporting Summary [file 41467_2025_66072_MOESM2_ESM.pdf]

nature portfolio

Corresponding author(s): Marta Gonzalaz

Last updated by author(s): Mar 11, 2025

Reporting Summary

Nature Portfolio wishes to improve the reproducibility of the work that we publish. This form provides structure for consistency and transparency in reporting. For further information on Nature Portfolio policies, see our Editorial Policies and the Editorial Policy Checklist.

Statistics

For all statistical analyses, confirm that the following items are present in the figure legend, table legend, main text, or Methods section.

n/a	Confirmed	
x		The exact sample size (n) for each experimental group/condition, given as a discrete number and unit of measurement
x		A statement on whether measurements were taken from distinct samples or whether the same sample was measured repeatedly
x		The statistical test(s) used AND whether they are one- or two-sided<br>*Only common tests should be described solely by name; describe more complex techniques in the Methods section.*
x		A description of all covariates tested
x		A description of any assumptions or corrections, such as tests of normality and adjustment for multiple comparisons
x		A full description of the statistical parameters including central tendency (e.g. means) or other basic estimates (e.g. regression coefficient) AND variation (e.g. standard deviation) or associated estimates of uncertainty (e.g. confidence intervals)
x		For null hypothesis testing, the test statistic (e.g. F, t, r) with confidence intervals, effect sizes, degrees of freedom and P value noted<br>*Give P values as exact values whenever suitable.*
x		For Bayesian analysis, information on the choice of priors and Markov chain Monte Carlo settings
x		For hierarchical and complex designs, identification of the appropriate level for tests and full reporting of outcomes
x		Estimates of effect sizes (e.g. Cohen's d, Pearson's r), indicating how they were calculated

Our web collection on statistics for biologists contains articles on many of the points above.

Software and code

Policy information about availability of computer code

Data collection	Data collection code described in https://zenodo.org/records/17199127
Data analysis	Data analysis code is described in https://zenodo.org/records/17199127

For manuscripts utilizing custom algorithms or software that are central to the research but not yet described in published literature, software must be made available to editors and reviewers. We strongly encourage code deposition in a community repository (e.g. GitHub). See the Nature Portfolio guidelines for submitting code & software for further information.

Data

Policy information about availability of data

All manuscripts must include a data availability statement. This statement should provide the following information, where applicable:

- Accession codes, unique identifiers, or web links for publicly available datasets
- A description of any restrictions on data availability
- For clinical datasets or third party data, please ensure that the statement adheres to our policy

Data is available at https://doi.org/10.5281/zenodo.17190814

Research involving human participants, their data, or biological material

Policy information about studies with human participants or human data. See also policy information about sex, gender (identity/presentation), and sexual orientation and race, ethnicity and racism.

Reporting on sex and gender	Sex and gender were not considered in the study design
Reporting on race, ethnicity, or other socially relevant groupings	Race, ethnicity or other socially relevant groupings are not considered in study design
Population characteristics	No human participants
Recruitment	No participants were recruited in this study
Ethics oversight	No information on the approval of the study is available

Note that full information on the approval of the study protocol must also be provided in the manuscript.

Field-specific reporting

Please select the one below that is the best fit for your research. If you are not sure, read the appropriate sections before making your selection.

☐ Life sciences ☒ Behavioural & social sciences ☐ Ecological, evolutionary & environmental sciences

For a reference copy of the document with all sections, see nature.com/documents/nr-reporting-summary-flat.pdf

Behavioural & social sciences study design

All studies must disclose on these points even when the disclosure is negative.

Study description	A quantitative study to understand electric vehicle adoption.
Research sample	We use publicly available datasets of electric vehicle purchase records and census tract–level statistics (vehicle counts and income) from Washington and California. We selected these states for their high EV penetration and policy relevance.
Sampling strategy	The study uses full samples from public administrative and census datasets, with no additional sampling procedures. Sample sizes were determined by data availability and are sufficient for robust analysis; qualitative sampling criteria do not apply.
Data collection	The data were obtained from publicly available datasets retrieved online. The researchers of this study were not involved in data recording or production. No direct participants or researchers were present during data generation, and issues of blinding are not applicable.
Timing	The Electric Vehicle Title and Registration Activity dataset covers 2010–2022. The California Air Resources Board Clean Vehicle Rebate Project spans 2010–2022. The U.S. Census Bureau data were collected between 2019–2020.
Data exclusions	No data were excluded in this study.
Non-participation	No participants were recruited in this study.
Randomization	This study does not involve experimental groups or direct participant allocation. All analyses are based on publicly available datasets retrieved online, and the researchers were not involved in data collection or dataset production.

Reporting for specific materials, systems and methods

We require information from authors about some types of materials, experimental systems and methods used in many studies. Here, indicate whether each material, system or method listed is relevant to your study. If you are not sure if a list item applies to your research, read the appropriate section before selecting a response.

Materials & experimental systems

n/a	Involved in the study
☒	☐ Antibodies
☒	☐ Eukaryotic cell lines
☒	☐ Palaeontology and archaeology
☒	☐ Animals and other organisms
☒	☐ Clinical data
☒	☐ Dual use research of concern
☒	☐ Plants

Methods

n/a	Involved in the study
☒	☐ ChIP-seq
☒	☐ Flow cytometry
☒	☐ MRI-based neuroimaging

Plants

Seed stocks	No seed stocks or plants were used in this study
Novel plant genotypes	No novel plant genotypes were used in this study
Authentication	No authentication procedure were used in this study
